# Equity in evidence synthesis: You can't play on broken strings

**DOI:** 10.1002/cesm.12091

**Published:** 2024-06-18

**Authors:** Tamara Lotfi, Vivian Welch, Jordi P. Pardo, Jennifer Petkovic, Shaun Treweek, Andrea J. Darzi, Rebecca Glover, Declan Devane, Meera Viswanathan, Lawrence Mbuagbaw, Kevin Pottie, Elizabeth Kristjansson, Shahab Sayfi, Lara Maxwell, Olivia Magwood, Damian Francis, Dru Riddle, Beverly Shea, Peter Tugwell

**Affiliations:** ^1^ Department of Health Research Methods, Evidence & Impact McMaster University Hamilton Ontario Canada; ^2^ Bruyere Research Institute Ottawa Ontario Canada; ^3^ School of Epidemiology and Public Health University of Ottawa Ottawa Ontario Canada; ^4^ Department of Medicine, Faculty of Medicine University of Ottawa Ottawa Ontario Canada; ^5^ Ottawa Hospital Research Institute Ottawa Ontario Canada; ^6^ Faculty of Medicine University of Ottawa Ottawa Ontario Canada; ^7^ Health Services Research Unit University of Aberdeen Aberdeen UK; ^8^ Michael G. DeGroote National Pain Center McMaster University Hamilton Ontario Canada; ^9^ Department of Anesthesia, Faculty of Health Sciences McMaster University Hamilton Ontario Canada; ^10^ Department of Health Services Research and Policy LSHTM London UK; ^11^ School of Nursing and Midfwifery University of Galway Galway Ireland; ^12^ Evidence Synthesis Ireland & Cochrane University of Galway Galway Ireland; ^13^ RTI International Durham North Carolina USA; ^14^ Department of Pediatrics McMaster University Hamilton Ontario Canada; ^15^ Biostatistics Unit, Father Sean O'Sullivan Research Centre, St Joseph's Healthcare Hamilton Ontario Canada; ^16^ Centre for Development of Best Practices in Health (CDBPH), Yaoundé Central Hospital Yaoundé Cameroon; ^17^ Division of Epidemiology and Biostatistics, Department of Global Health Stellenbosch University Cape Town South Africa; ^18^ Research Chair in Family Medicine Dalhousie University Halifax Nova Scotia Canada; ^19^ School of Psychology University of Ottawa Ottawa Ontario Canada; ^20^ Interdisciplinary School of Health Sciences University of Ottawa Ottawa Ontario Canada; ^21^ School of Health and Human Performance, Center for Health and Social Issues, Georgia College Milledgeville Georgia USA; ^22^ Texas Christian University Fort Worth Texas USA; ^23^ Cochrane US Network Portalnd Oregon USA; ^24^ Department of Medicine, Faculty of Medicine, Bruyère Research Institute University of Ottawa Ottawa Ontario Canada; ^25^ Ottawa Hospital Research Institute, Clinical Epidemiology Program Ottawa Ontario Canada

In the 2022 Cochrane Lecture [1], Jimmy Volmink, recognized as a pioneer of evidence‐based medicine in Africa, challenged Cochrane to enhance its equity efforts and suggested five ways to do so. We, as members of the Campbell and Cochrane Health Equity Thematic group*, fully agree with his suggestions and have developed an actionable plan, described below. We invite the global community to join us in our efforts to meet the equity gaps in research and practice.

Population health and healthcare delivery should be equitable and the research that guides it equity sensitive. By this, we mean that we need to focus on the distribution of health outcomes in the population not just overall health. That is, people should have equal opportunities for health and are not subjected to systemic discrimination or structural barriers to health. It is an ambitious goal and one that many of us who work in healthcare delivery and health research are striving for.

This includes those of us who work in evidence synthesis. Synthesizers of other researchers' evidence may think that our handling of equity cannot be better than the handling of equity in the research we synthesize. We, as members of the Campbell and Cochrane Health Equity Thematic Group, disagree. To truly address inequity, evidence synthesis must take into account equity considerations in a systematic and explicit manner, regardless of how equity was addressed in the original research. We believe that evidence synthesis should lead the way in promoting equity, rather than simply reflecting the approaches taken in the primary research that is included in our reviews (Box 1).

Box 1 **– Analogy between the bass instrument and evidence synthesis. *(Image from* https://commons.wikimedia.org/wiki/File:Bass_Guitar.svg)**

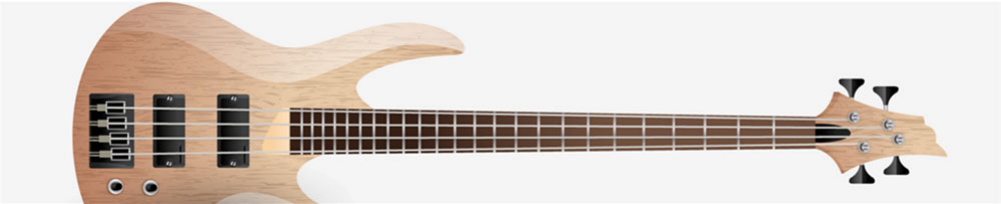

Drawing an analogy with music, consider the bass guitar (Box 1). As part of a band, the bass follows a predefined and structured melody. Similarly, evidence synthesis is rigorously produced by a team to respond to a need for a particular audience (patient important question, clinical practice gap, or policy decision).

The overall tone of a chord in a melody depends on the quality, integrity, and tuning of the strings. In the realm of research, these strings symbolize four crucial factors: the importance of the research question, the methodological rigor, adherence to international reporting standards, and, importantly, the adoption of an equity lens. Systematic reviews that are deficient in any of these four factors is more likely to contribute to research waste than be beneficial: you can't play on broken strings.

## 1 ACTIONABLE PLAN IN RESPONSE TO PROF. VOLMINK

We fully agree that Cochrane cannot succeed in better addressing health equity in systematic reviews without also addressing inequities in its own organization and governance. As members of the Campbell and Cochrane Health Equity Thematic group, we commit to the following actions.

### 1.1 Set priorities for reviews based on health equity and global burden of disease

Prof Volmink found that only 1.3% (113) of Cochrane reviews directly address the social determinants of health, social factors and issues of health equity, poverty, racism, and sexism [1]. Furthermore, the top 25 conditions that contribute to the greatest number of Disability‐Adjusted Life Years (DALYs) are addressed in less than a quarter of Cochrane reviews (19.2%) [1].

The Campbell and Cochrane Health Equity Thematic group is currently supporting the prioritization of review questions in collaboration with the Cochrane Commissioning Initiative. This prioritization process considers health equity and Global Burden of Disease as key criteria. For health equity, we use the PROGRESS Plus framework [2] as a guide. This framework addresses **P**lace of residence, **R**ace/ethnicity/culture/ancestry/language, **O**ccupation, **G**ender, **R**eligion, **E**ducation, **S**ocio‐economic status, **Social** capital, and “Plus” that entails age, sexual orientation and disability [2, 3, 4]. In the prioritization process, we are also considering the intersectionality of the barriers and opportunities that one could face across these factors. We have also initiated a process for similar equity‐focused prioritization with the Cochrane Nutrition and Physical Activity Thematic group. The prioritization process will be continuous to reflect the changing gaps/needs for evidence and disease occurrence. To ensure that this process is inclusive, it will incorporate input from interest‐holders globally through existing evidence synthesis groups, consumer networks, and other users.

### 1.2 We will advocate and support initiatives to promote equity, diversity, and inclusion in Cochrane

Prof Volmink found that only 0.3% of Cochrane authors are from a low‐income country, 3.3% from a lower‐middle‐income country, and 12.7% from an upper‐middle‐income country [1]. Others have reported similar low percentages of authors from low‐ and middle‐income countries (LMICs) [5, 6].

To address diversity in Cochrane review author teams, Cochrane must take the lead in driving change. It has already initiated this process with leading a commission and report on diversity and inclusion in Cochrane [7]. As above, Cochrane is committed to prioritizing equity‐focused reviews.

The Campbell and Cochrane Equity Thematic group will actively support initiatives to improve diversity and inclusion within Cochrane. We offer support and mentorship for equity‐focused reviews as part of our mandate and invite authors from LMICs to contact us when planning an equity‐focused review. We actively advocate for further reduction in registration fees and increased numbers of LMIC stipends at Cochrane Colloquia (around 40% of the early bird price [8]) and that these should be graded based on country income level.

### 1.3 We are actively seeking global representation in the governance of the Campbell and Cochrane Health Equity Thematic Group

Prof Volmink rightly pointed out that the majority of Campbell and Cochrane Equity Thematic group fellows and co‐convenors are based in Canada.

The Campbell and Cochrane Health Equity Thematic group recruited Lawrence Mbuagbaw from Cameroon, Gabriel Rada from Chile, and Ashrita Saran from India in 2022 [9]. We have developed a recruitment strategy to actively pursue global representation within our leadership team and working groups. We will draw on insight from successful examples of global collaboration within Cochrane and other relevant organizations (e.g., The International Clinical Epidemiology Network (INCLEN) [10], the Global Evidence Synthesis Initiative [GESI]), and aim to foster equitable global collaborations. Such collaborations could share common goals, such as making the evidence available and accessible for those taking action towards the Sustainable Developmental Goals [11]. Though our current process of recruitment is mainly targeted, we will actively promote a call for interest through the Regional Cochrane Centers and Networks to engage other researchers working in health equity. This means that we will ensure equitable opportunities for diverse leadership roles across our group's projects, guided by terms of reference that outlines our values, roles, expectations, and models for recognizing contributions (including provision for paid time for contributions for authors in LMIC).

### 1.4 Call for action: Turning the volume up

All the strings of a bass guitar matter equally to generate the planned melody. We strongly advocate for the integration of health equity considerations in systematic reviews so that decision makers can be empowered to make equitable policies and take informed decisions. We need actions from all those involved in the research ecosystem; for example, journal editors need to require better equity reporting, funders need to insist on equity assessment and equity, diversity, and inclusion (EDI) within research teams, and researchers need to use and develop equity‐focused tools to better document implications for health equity in their research.

You can't play on broken strings. Let's fix the equity string together‐please get in touch with us at: https://www.cochrane.org/about-us/our-global-community/thematic-groups/health-equity and join us at the Global Evidence Summit in Prague, September 2024.

*The Campbell and Cochrane Equity Thematic group is one of the six thematic groups announced by Cochrane as part of its new direction [12]. *We aim to encourage deeper consideration of health equity across Cochrane, building on existing training material and prior research. We have five objectives for the next 5 years, which include: (1) Promote equity in the health evidence base, (2) Ensure equitable processes for stakeholder engagement, (3) Produce high‐priority, equity‐focused evidence syntheses, (4) Build capacity for equity design, analysis, and reporting, and (5) Promote equity in implementation tools*.

## AUTHOR CONTRIBUTIONS

**Tamara Lotfi**: Conceptualization, writing—original draft, reviewing and editing. **Vivian Welch**: Conceptualization, writing—original draft, review & editing. **Jordi P. Pardo**: Conceptualization, writing—original draft, review & editing. **Jennifer Petkovic**: Conceptualization, writing—original draft, review & editing. **Shaun Treweek**: Writing—review & editing. **Andrea Darzi**: Writing—review & editing. **Rebecca Glover**: Writing—review & editing. **Declan Devane**: Writing—review & editing. **Meera Viswanathan**: Writing—review & editing. **Lawrence Mbuagbaw**: Writing—review & editing. **Kevin Pottie**: Writing—review & editing. **Elizabeth Kristjansson**: Writing—review & editing. **Shahab Sayfi**: Writing—review & editing. **Lara Maxwell**: Writing—review & editing. **Olivia Magwood**: Writing—review & editing. **Damian Francis**: Writing—review & editing. **Dru Riddle**: Writing—review & editing. **Beverly Shea**: Writing—review & editing. **Peter Tugwell**: Conceptualization, writing—original draft, review & editing.

## CONFLICT OF INTEREST STATEMENT

JPP is a member of the Cochrane Governing Board. The remaining authors declare no conflict of interest.

## Data Availability

Data sharing is not applicable to this article as no data sets were generated or analysed during the current study.
